# Safe and effective two-in-one replicon-and-VLP minispike vaccine for COVID-19: Protection of mice after a single immunization

**DOI:** 10.1371/journal.ppat.1009064

**Published:** 2021-04-21

**Authors:** Alexandru A. Hennrich, Bevan Sawatsky, Rosalía Santos-Mandujano, Dominic H. Banda, Martina Oberhuber, Anika Schopf, Verena Pfaffinger, Kevin Wittwer, Christiane Riedel, Christian K. Pfaller, Karl-Klaus Conzelmann

**Affiliations:** 1 Max von Pettenkofer Institute Virology, and Gene Center, LMU Munich, Munich, Germany; 2 Department of Veterinary Medicine, Paul-Ehrlich-Institute, Langen, Germany; 3 Institute of Virology, Department of Pathobiology, University of Veterinary Medicine Vienna, Vienna, Austria; Johns Hopkins University Bloomberg School of Public Health, UNITED STATES

## Abstract

Vaccines of outstanding efficiency, safety, and public acceptance are needed to halt the current SARS-CoV-2 pandemic. Concerns include potential side effects caused by the antigen itself and safety of viral DNA and RNA delivery vectors. The large SARS-CoV-2 spike (S) protein is the main target of current COVID-19 vaccine candidates but can induce non-neutralizing antibodies, which might cause vaccination-induced complications or enhancement of COVID-19 disease. Besides, encoding of a functional S in replication-competent virus vector vaccines may result in the emergence of viruses with altered or expanded tropism. Here, we have developed a safe single round rhabdovirus replicon vaccine platform for enhanced presentation of the S receptor-binding domain (RBD). Structure-guided design was employed to build a chimeric minispike comprising the globular RBD linked to a transmembrane stem-anchor sequence derived from rabies virus (RABV) glycoprotein (G). Vesicular stomatitis virus (VSV) and RABV replicons encoding the minispike not only allowed expression of the antigen at the cell surface but also incorporation into the envelope of secreted non-infectious particles, thus combining classic vector-driven antigen expression and particulate virus-like particle (VLP) presentation. A single dose of a prototype replicon vaccine complemented with VSV G, VSVΔG-minispike-eGFP (G), stimulated high titers of SARS-CoV-2 neutralizing antibodies in mice, equivalent to those found in COVID-19 patients, and protected transgenic K18-hACE2 mice from COVID-19-like disease. Homologous boost immunization further enhanced virus neutralizing activity. The results demonstrate that non-spreading rhabdovirus RNA replicons expressing minispike proteins represent effective and safe alternatives to vaccination approaches using replication-competent viruses and/or the entire S antigen.

## Author summary

Two critical problems are associated with replicating paramyxo- and rhabdovirus vaccines expressing SARS-CoV-2 spike (S) protein such as VSVΔG(S). One is eliciting of potentially disease-enhancing non-neutralizing antibodies, the other the S-mediated spread in humans. In view of the multi-organ tropism of SARS-CoV-2 in humans, their pathogenic outcome is not predictable. Here, we address and resolve both issues. We describe an innovative VSV vaccine, which is safe both in terms of virus propagation and immune response, as it is a non-spreading single round replicon vector, and the immunogen is limited to the spike’s receptor binding domain (RBD), which emerged as the antigen eliciting the desired virus-neutralizing antibodies in humans. An excellent protective immune response in animals is achieved by the design of a chimeric RBD-minispike, which allows a combined “2-in-1” approach, meaning that the optimized antigen is simultaneously presented on cells and on noninfectious virus-like particles (VLPs). With such enhanced RBD antigen presentation it is thus not necessary to use replication-competent virus or entire S antigen.

## Introduction

The current COVID-19 pandemic, caused by SARS-CoV-2, has claimed more than 2.7 million lives so far and represents an exceptional challenge for our society, economy, and science. Because of high morbidity and mortality in risk groups and possible long-term multi-organ sequelae, strategies to achieve sufficient natural herd immunity are not acceptable. We are therefore witnessing unprecedented efforts to develop vaccines to be administered to the majority of humanity. While it fortunately turns out that some approved SARS-CoV-2 vaccines can stimulate immune responses protecting from COVID-19 disease without overt immediate side effects and fundamentally contribute to future containment of the pandemic (see e.g. [1–4]), numerous and diverse vaccine candidates are being developed to meet the need for rapid protection of humans of all ages and conditions and/or preventing virus transmission. Prudent and transparent assessment of antigens, adjuvants and delivery vehicles is critical to prevent medical hazards and to inspire public confidence in vaccines.

Of particular concern for vaccine safety are potentially precarious delivery vehicles, including newly developed replicating viruses as well as harmful immune responses to inadequate antigens, known as antibody-dependent enhancement (ADE). Of special concern in case of respiratory viruses like SARS-CoV-2 is vaccine-associated enhanced respiratory disease (VAERD) [5–7], which happened previously after vaccination with conformationally incorrect viral antigens of respiratory syncytial virus (RSV). Especially, VAERD was associated with high levels of non-neutralizing antibodies. A combination of immune complex deposition, complement activation, and Th2-biased immune response led to enhancement of respiratory symptoms [8–10].

The pandemic SARS-CoV-2 is a betacoronavirus [11–13] closely related to the severe acute respiratory syndrome (SARS) virus (now named SARS-CoV-1) which emerged in 2003 [14,15]. Previous research on SARS-CoV-1 was highly instructive and provided valuable blueprints for the development of COVID-19 vaccines. In particular, Buchholz and colleagues showed that the viral surface spike (S) protein is the only virus protein that stimulates virus neutralizing antibodies (VNAs) [16], which are crucial for most vaccine approaches. Accordingly, S is the main target of current COVID-19 vaccines and vaccine candidates [17] and VNAs are established in the meantime as the main correlate of protection after infection or vaccination against COVID-19 in humans and animal models [18–22].

The class I transmembrane protein S is the primary determinant of coronavirus tropism and transmission. The S precursor protein is processed by cellular proteases into the mature N-terminal S1 and the membrane-bound S2 subunits [23–25]. S1 contains the receptor-binding domain (RBD) responsible for attachment of the virus to the main cellular receptor, angiotensin-converting enzyme 2 (ACE2) [26,27]. Binding of the RBD to the receptor results in profound structural rearrangements required for membrane fusion by the S2 subunit, and release of the viral RNA genome into the cytoplasm. Molecular differences to SARS-CoV-1 S include a higher binding affinity of the RBD to the ACE2 molecule [26–28] and the presence of a multibasic cleavage site, probably promoting proteolytic maturation and transport of the protein [11,23,24]. These factors likely contribute to an extended host and organ range and the high contagiousness of SARS-CoV-2 [29–31].

As accumulating data show, COVID-19 patients readily develop high levels of antibodies directed against the entire S protein, most of which, however, do not neutralize virus infectivity. In contrast, the overwhelming amount of RBD-binding antibodies exhibits neutralizing activity [22,32–35]. Of note, non-neutralizing antibody epitopes of SARS-CoV-1 and SARS-CoV-2 S proteins were found to enhance virus infection *in vitro* [36,37] and it was suggested that anti-S IgG from severely ill COVID-19 patients may promote hyper-inflammatory responses [38]. Focusing on the RBD immunogen in order to elicit potent neutralizing antibodies and to avoid unnecessary or potentially harmful non-neutralizing S antibodies is therefore advisable.

Recombinant negative strand RNA viruses including rhabdoviruses like the animal pathogen VSV [39] or the zoonotic rabies virus [40] are attractive platforms for experimental vaccines against emerging and neglected viral diseases, as well as for oncolytic immune therapies (for recent reviews see [41,42]). Rhabdoviruses are bullet shaped, cytoplasmic, and non-integrating RNA viruses encoding a single glycoprotein (G) responsible for receptor attachment and infection of cells. As illustrated before, VSV full-length or G gene-deficient (VSVΔG) vectors expressing functional S of SARS-CoV-1 induced a protective immune response in animal models [43,44]. As residual pathogenicity of recombinant full length VSV is largely attributed to the glycoprotein G [45], one strategy to attenuate VSV vaccines is the replacement of the G gene by those of heterologous envelope proteins, as exemplified in the recently approved Ebola vaccine VSV-Zebov (Ervebo) [46]. Not surprisingly, G-deficient VSV expressing fully functional SARS-CoV-2 S proteins have rapidly been prepared and proposed as COVID-19 vaccine candidates [47–51]. Importantly, and in contrast to SARS-CoV-1, the authentic SARS-CoV-2 spike protein can readily mediate spread and amplification of S surrogate VSVs in cell culture, organoids, and animals [43,44,52]. Moreover, VSVΔG-SARS-CoV-2 S rapidly developed mutations in the S gene to adapt to cell culture conditions and to yield high titer viruses, as well as antibody escape mutations [47,53,54]. As attenuation of VSV evidently depends on the glycoproteins used for construction of surrogate viruses and their tropism [55], extensive preclinical testing is required—as was done in the case of VSV-Zebov (for review see) [46]—to inspire confidence in any replicating VSV or VSVΔG surrogate virus vaccine.

Here we propose a safe and highly effective alternative to both replication competent viruses and expression of the full-length SARS-CoV-2 S antigen to minimize potentially detrimental immune responses. Using structure-guided design, we developed a chimeric transmembrane RBD construct, termed “minispike”, for enhanced and structurally correct antigen presentation. In the minispike construct, the RBD domain is fused to a C-terminal transmembrane stem-anchor of the G protein of rabies rhabdovirus (RABV), to allow effective expression as a cell-membrane-bound immunogen. In addition, expression of the minispike from spreading-deficient (G-deficient) VSV or RABV replicon vectors results in the secretion of non-infectious VLPs decorated with the minispike antigen. Notably, immunization with a single dose of a G-complemented VSV replicon encoding a single copy of the RBD minispike gene (VSVΔG-minispike-eGFP) was found to protect transgenic K18-hACE2 mice from disease. As the minispike construct is compatible with RABV, VSV and probably other rhabdoviruses, which all are amenable to envelope switching, the rhabdovirus minispike system offers attractive options for a diversity of prime/boost regimens, including oral immunization with RABV G complemented viruses.

## Results

### Design of a rhabdovirus RBD-minispike

**Fig 1:**
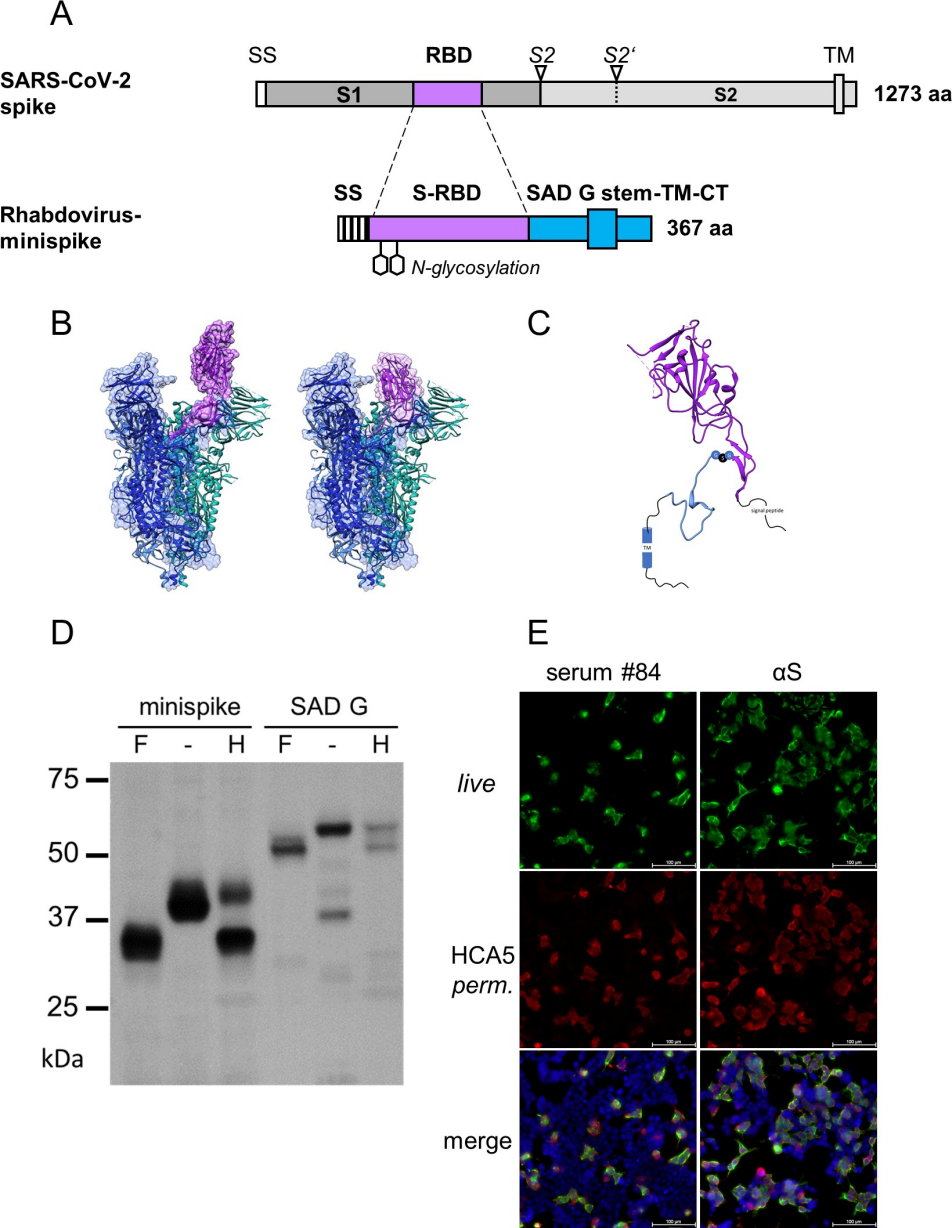
Design and expression of minispike. (A) Schematic representation of the SARS-CoV-2 spike protein and of the chimeric minispike protein containing a hIgG signal sequence (SS) the SARS-CoV-2 RBD (purple), and the RABV G stem/anchor sequence (blue). Two consensus N-gylcosylation sites are indicated. S2 and S2′ arrowheads indicate protease cleavage sites, TM transmembrane domain. (B) Ribbon model of the SARS-CoV-2 S protein in the RBD “up” (PDB 6VYB) and “down” (PDB 6VXX) conformation with RBD residues included in the minispike protein highlighted in purple. The EM density map is shown in grey. (C) Model of the chimeric minispike construct. Elements with available structural information are shown as ribbon diagrams and include the RBD of SARS-CoV-2 (purple, PDB 6VXX) and parts of the RABV G-protein (blue, PDB 6LGX). The GSG Linker connecting the two domains is depicted as blue (G) and black (S) circles. Elements of unknown structure including signal peptide and C-terminus of RABV G are shown as black lines. A blue cylinder (TM) indicates the transmembrane domain. (D) Complex N glycosylation of minispike protein. Extracts from HEK293T cells transfected with pCR3-minispike and RABV G as a control were treated with PNGase F (+F), which cleaves off all N-linked oligosaccharides, left untreated (-) or treated with Endoglycosidase H (+H), unable to cleave complex sugars. The minispike protein acquires EndoH-resistant complex sugars, indicating transport through the Golgi apparatus. Proteins were visualized by incubation with HCA-5 serum, recognizing the common C-tail. (E) Surface expression and recognition of minispike by COVID-19 patient serum. Live, unpermeabilized HEK293T cells transfected with pCR3-minispike were first stained with a representative COVID-19 convalescent serum at 1:300 dilution (left panel) or conformation-specific SARS-CoV S Mab CR3022 (right panel), and anti-human IgG/AlexaFluor488 (green). Following fixation with 4% paraformaldehyde (PFA) and permeabilization with 0,1% Saponine, cells were in addition stained with HCA-5/anti-rabbit AlexaFluor555 (red) recognizing the intracellular RABV C-tail. Cell nuclei were visualized with ToPro3-iodide (blue). Size bar indicates 100 μM.

The RBD of SARS-CoV-2 spike protein was identified by sequence homology to the SARS-CoV-1 RBD and by functional studies [26,28,56,57]. Structural analyses revealed an autonomously folding, discrete globular-shaped domain, able to switch between “up” and “down” configurations in the context of the pre-fusion form of the S protein, and in which the up-conformation is needed to engage the ACE2 receptor [27,58]. Based on the structure analysis we selected residues 314–541 (QTSN…KCVNF) to be included in a chimeric transmembrane minispike in which the RBD domain is presented in a natural conformation. In addition, the minispike was designed to be compatible for presentation on the cell membrane as well as for its incorporation into the envelope of rhabdovirus-like particles, including VSV and RABV (Fig 1A–1C).

The amino-terminal signal peptide from human IgG heavy chain (*Ig G HV 3–13*) was used to promote translation into the endoplasmic reticulum. The carboxy-terminus of the RBD sequence was fused via a short synthetic linker to a transmembrane stem-anchor derived from the glycoprotein of the RABV strain SAD, containing the membrane proximal part of the G ectodomain (stem), the trans-membrane domain, and the cytoplasmic sequence of SAD G [59]. The entire construct comprises 367 amino acid residues, including the signal sequence, and two N-glycosylation sites in the RBD moiety (NITNLCPFGEVFNAT). The SAD G stem was selected because it should allow incorporation into the envelopes of not only RABV, but also of non-RABV rhabdoviruses, such as VSV, which has less stringent sequence requirements for membrane protein incorporation [60,61]. In the case of VSV, the heterologous stem-anchor was predicted not to critically compete with VSV G incorporation needed during production of infectious single cycle VSV replicon viruses.

Expression of the minispike construct in HEK293T cells after transfection with plasmid-encoded minispike (pCR3-minispike) was at first analyzed by Western blot with an anti-SAD C-tail peptide serum (HCA-5) recognizing the RABV-derived anchor sequence (Fig 1D). Minispike proteins were of the predicted molecular weight range, and deglycosylation experiments with PNGase F and Endo-H confirmed the presence of complex sugar chains, indicating correct processing and transport through the Golgi apparatus. Expression at the cell surface was further demonstrated by microscopic imaging (Fig 1E). Positive staining of transfected unfixed live cells with serum from convalescent COVID-19 patients as well as with the RBD antibody CR3022, which in the context of the S protein binds to an epitope of the RBM only accessible in the up conformation [21,62] indicated that the minispike RBD acquires a conformation corresponding to that of the natural SARS-CoV-2 RBD.

### Construction of minispike-expressing rhabdoviruses

**Fig 2:**
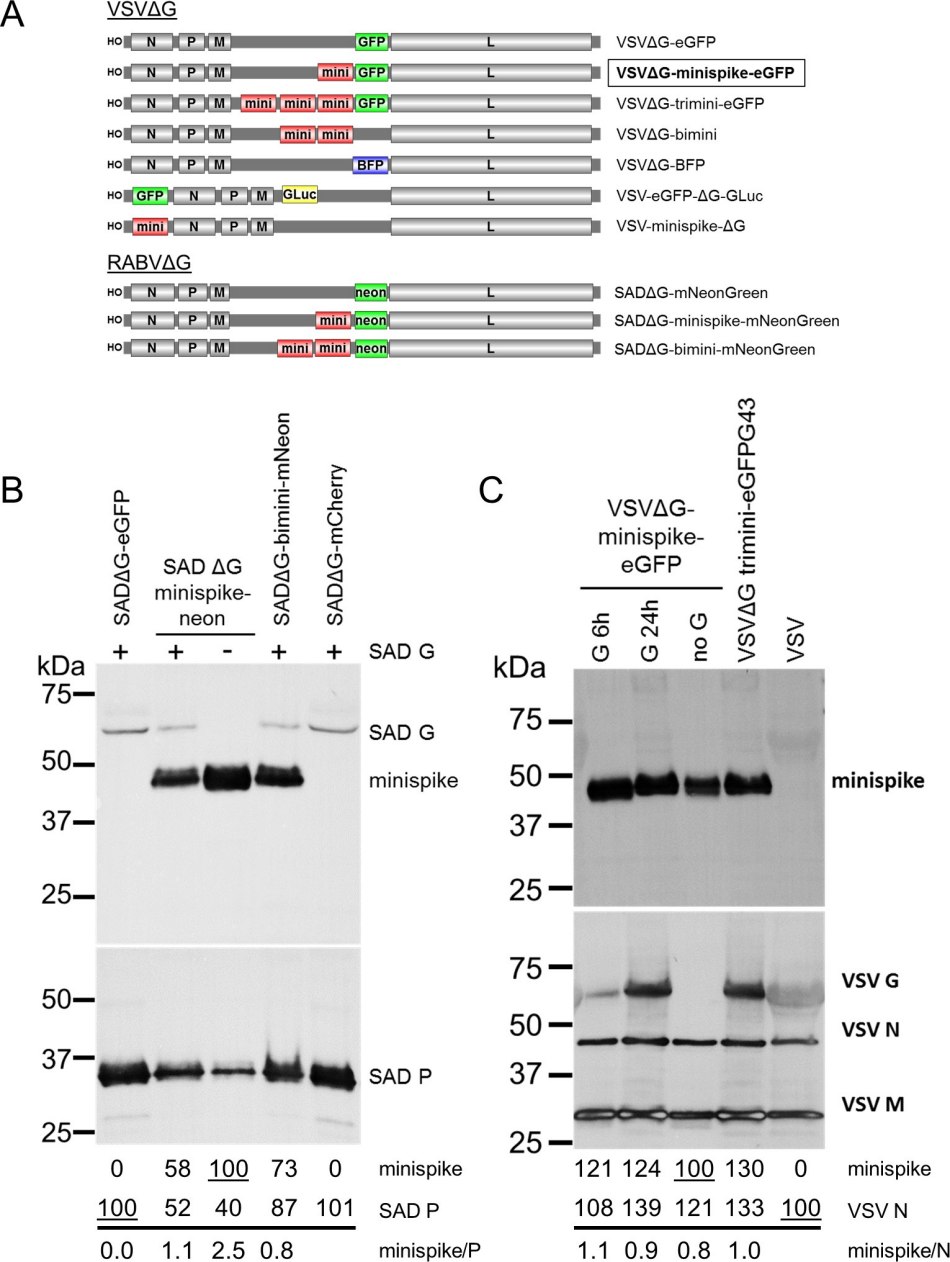
Characterization of minispike rhabdoviruses. (A) Schematic of VSVΔG and RABVΔG constructs used here. (B) Incorporation of minispike in RABV SAD envelopes. Cell-free SADΔG-virus particles as indicated were generated in HEK293T cells in the absence or presence of RABV SAD G. Lanes were loaded with 1 million infectious units each, blots were incubated with HCA-5 recognizing the cytoplasmic tails of minispike and SAD G (upper panel) and anti-RABV P serum to determine virus load (lower panel). Quantification of band intensities indicates competition of minispike and SAD G for incorporation into virions. (C) Incorporation of minispike in VSV envelopes. Cell-free minispike-encoding VSVΔG viruses were generated in HEK293T cells expressing VSV G from transfected pCAGGS-VSV G for 6 or 24 hrs prior to infection, or in stable BHK-G43 cells (VSVΔG-trimini-eGFP) induced at the time of infection were purified by ultracentrifugation. Lanes were loaded with 1 million infectious units of G-containing infectious viruses, and the same volume of non-infectious viruses (no G). Blots were incubated with serum HCA-5 recognizing the RABV G-derived C-tail of the minispike (upper panel) or with anti-VSV serum recognizing viral N, M, and G proteins (lower panel). Note that G24h preparation contains G vesicles (see Fig 3).

Molecular clones of the Indiana strain of VSV (VSIV) [39] were used as a basis for generation of a series of G gene-deleted VSV replicons (VSVΔG) encoding the minispike (Fig 2A). The constructs included eGFP reporter viruses and viruses expressing single or multiple copies of the minispike gene inserted either upstream of the L gene, or at the 3’ proximal gene position, which in rhabdoviruses is transcribed most abundantly [63,64]. Recombinant viruses were rescued in HEK293T cells and propagated in cells transfected with VSV G plasmids or in a cell line expressing VSV G (BHK-G43) [65]. All VSVΔG viruses reached comparable titers in the range of 5x10^7^ to 3x10^8^ ffu/mL after 20–24 h of infection. G gene-deficient RABV cDNA and replicons were generated on the basis of SADΔG-eGFP and grown as described before [61,66–68].

### Generation of minispike VLPs and mosaic viruses

As the minispike stem-anchor is derived from the G protein of the RABV SAD strain, we first studied incorporation into virions of the autologous SADΔG-minispike-mNeonGreen and SADΔG-bimini-mNeonGreen (Fig 2B). Supernatant virions were concentrated by ultracentrifugation through a sucrose cushion and equivalent infectious units were processed for Western blot analysis with a RABV P serum, and a G C-tail serum to detect virus-associated minispikes and RABV G. Minispike was effectively incorporated into particles both in the absence and in presence of the parental SAD G. In the presence of SAD G less minispike was observed in RABV particles (Fig 2B), suggesting competition of the homologous SAD G and minispike for incorporation.

To examine incorporation of the “heterologous” minispike into VSV particles, VSVΔG-minispike-eGFP stocks were produced in cells transfected with VSV G expression plasmids. For preparation of one stock, VSV G was expressed only 6 hours before VSVΔG-minispike-eGFP infection occurred, in another preparation VSV G was allowed to accumulate to high levels for 24 hours before infection. Western blot analysis of 1 million infectious units of each with anti-VSV serum revealed effective incorporation along with VSV G (Fig 2C).

Rhabdovirus G proteins are incorporated into viral envelopes as G trimers which is driven by interaction of the C-tails with the internal M-coated viral RNP [69–71], and their incorporation supports virus budding [60,72]. Thus, the presence of minispike protein in VSV envelopes could be due to its co-incorporation with VSV G molecules as hetero-trimeric complexes. To determine whether RBD minispike alone supports budding of VSV VLPs, VSVΔG-minispike-eGFP stocks were produced in non-complementing cells and processed as above. The absence of VSV G did not prevent incorporation of the minispike (Fig 2C; lane “no G”), revealing autonomous incorporation and release of non-infectious minispike VSV VLPs from infected, non-complementing cells. Notably, comparable amounts of minispikes were observed in VSV particles irrespective of the presence of G (Fig 2C). As VSVΔG viruses encoding multiple minispike genes did not show improved minispike incorporation or infectious titers, the single copy VSVΔG-minispike-eGFP was chosen for further analyses.

**Fig 3:**
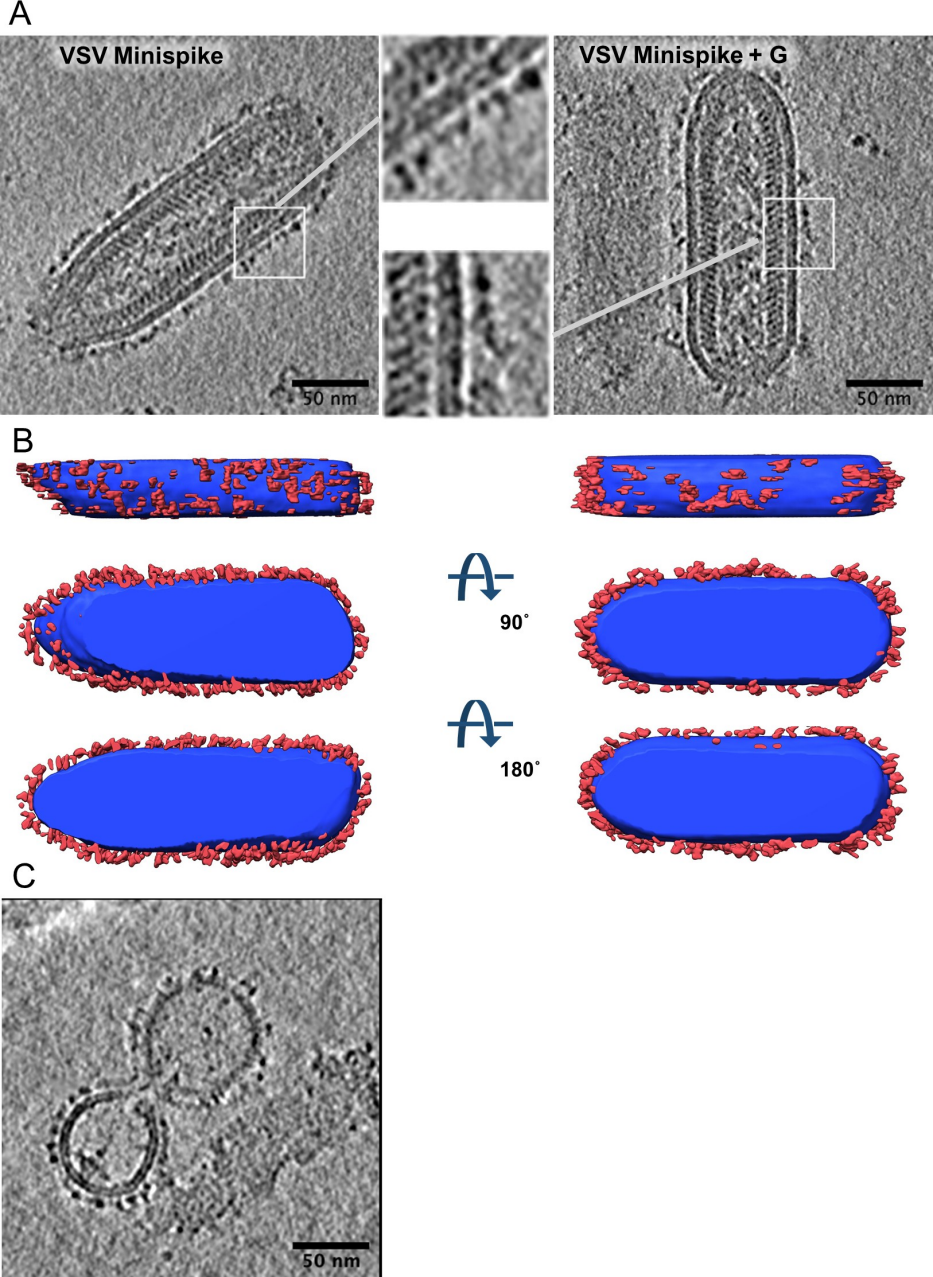
Characterization of minispike VLPs and mosaic viruses by cryo-EM. (A) Slices through cryo-electron tomograms of minispike-encoding VSV replicons generated in the presence of the autologous G proteins (right panel) or in the absence (left panel). Inserts show an enlarged membrane section as indicated by white squares. VSV particles are covered by a dense array of surface protrusions, which appears more heterogenous in the presence of G proteins (see S1 Fig for RABV particles and determination of length distribution). (B) Surface representation of densities of the virus particle corresponding to the membrane surrounded compartment (blue) and the surface glycoprotein layer (red). (C) VSVΔG preparations produced in the presence of VSV G contain both virions with mosaic envelope and G-coated non-viral vesicles (aka Gesicles).

The composition of viral envelopes was studied in more detail by cryo-electron tomography. In the absence of a rhabdovirus G protein, VSV (Fig 3) as well as RABV VLPs (S1 Fig) contained a homogenous surface glycoprotein layer, reflecting autonomous incorporation of the minispike as suggested by the above WB experiments. The size of the globular RBD is about 60 x 35Å [27,58]. The minispike construct should thus protrude between 6 and 11 nm from the membrane. The prefusion form of rhabdovirus G protein is protruding about 8.5 nm from the virus membrane, whilst the post-fusion form is protruding about 13 nm [73]. Measuring out RABV virions expressing only G or minispike, or the combination of both, revealed differences in length of the surface protrusions (S1 Fig). G-covered particles had surface proteins with an average length of 8.15 nm (n = 99, STD 1.07 nm) whilst in minispike VLPs this length was reduced to 7.70 nm (n = 77, STD 1.35). In the presence of both G and minispike, surface protein protrusions had an average length of 8.45 nm (n = 111, STD 1.47 nm). A direct morphological separation between G and minispike was not possible, and no higher order arrangement of the surface glycoproteins was discernible in the tomograms, suggesting random mixing.

Of note, virus preparations produced in the presence of VSV G contained non-viral vesicles with a homogenous, distinct surface protein layer (Fig 3C). They likely represent the typical ‘Gesicles’ or G-nanovesicles formed by the autonomous budding activity of the full length VSV G protein [74,75]. We did not observe similar vesicular structures after expression of RABV G or minispike. As for the parental RABV G, the chimeric minispikes thus lack the ability of efficient autonomous budding.

### VSV-expressed minispike is recognized by COVID-19 patient sera

**Fig 4:**
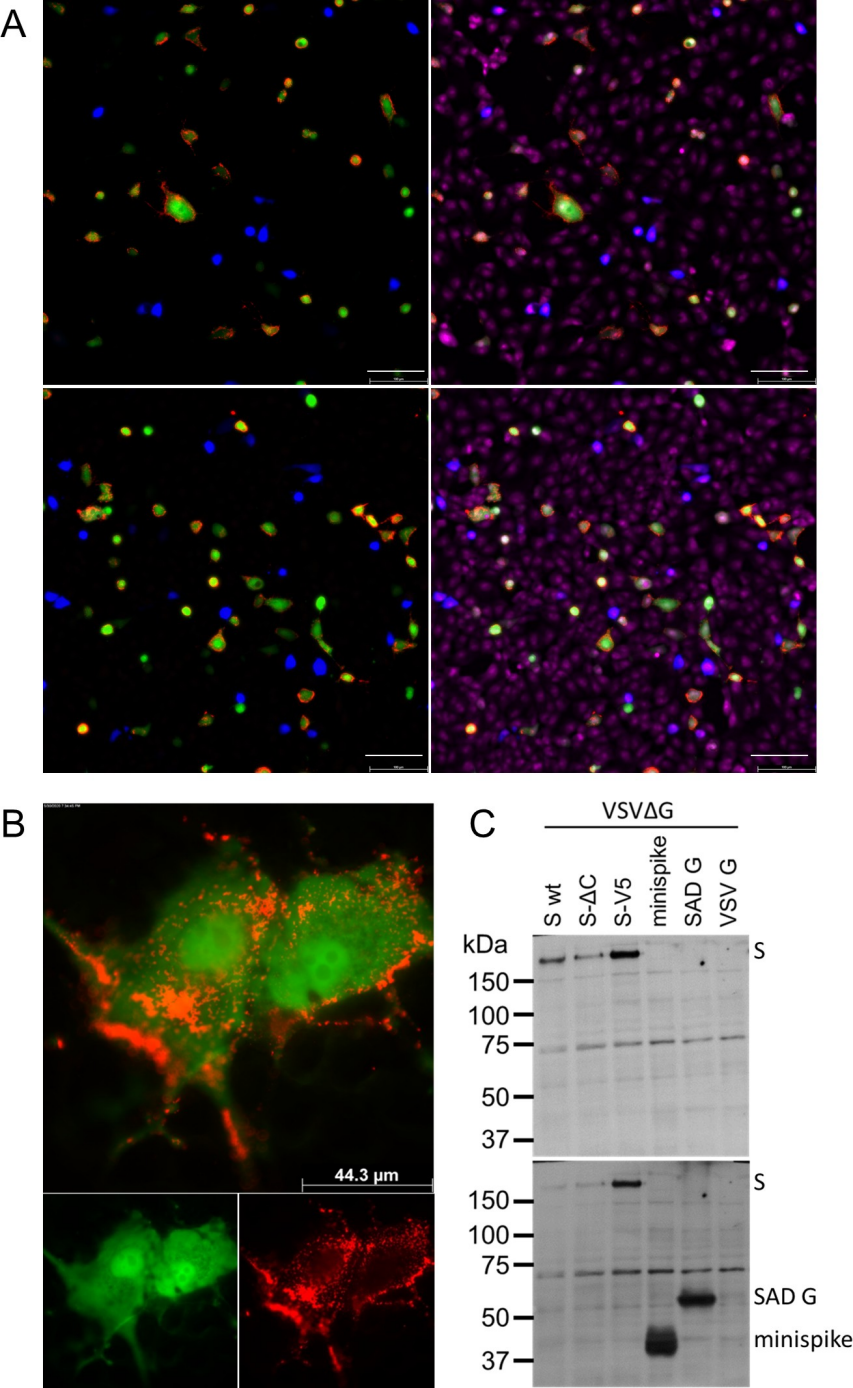
Virus minispike presents conformational RBD epitopes. (A) VeroE6 cells infected over night with VSVΔG-minispike-eGFP (green) are recognized by IgG of S ELISA-positive patients in contrast to a control replicon expressing blue fluorescent protein (VSVΔG-BFP). Live cells were incubated with patient serum (pat. ID 84; for other sera see S1 Fig) (upper panel) or SARS-Cov S Mab CR3022 visualized with anti-human IgG Alexa 555 (red). After permeabilization, nuclei were visualized with ToPro3-iodide. 200x magnification. Size bar represents 100 μm. (B) Cells infected with a RABV replicon expressing minispike (green) are recognized by IgG of S ELISA-positive patients (red). Live unpermeabilized VeroE6 cells were infected with SADΔG-minispike-mNeongreen, incubated over night at 37°C, and stained as described for (A), but without permeabilization and nuclear staining. 1000 x magnification. (C) Poor recognition of denatured minispike protein by patient immune sera. VSVΔG-eGFP virions pseudotyped with full length wt S, a C-terminally truncated S (SΔC) or a V5-tagged S protein (S-V5) were processed for denaturing SDS Western blot and probed with a representative patient serum (Pat. #84). In contrast to the full length S proteins, denatured minispike was not readily recognized by human serum IgG. In the lower panel minispike expression was controlled by additional incubation of the same blot with HCA-5 peptide serum recognizing the C-tail present in SAD G and minispike.

To corroborate that VSV replicons express correctly folded, processed and cell surface targeted RBD antigens, VeroE6 cells were infected with VSVΔG-minispike-eGFP (G) and, as a control, with a VSVΔG expressing only blue fluorescent protein (VSVΔG-tagBFP (G)), and probed with a collection of sera from patients previously tested positive for anti-S IgG in a commercial ELISA. EGFP and tagBFP fluorescence were used as controls to identify virus-infected cells, while bound patient IgG was detected with an Alexa555-labelled anti-human IgG secondary antibody. As illustrated in Fig 4A for a representative serum, ELISA-positive sera or Mab CR3022 brightly stained unfixed living cells infected with VSVΔG-minispike-eGFP, but not with VSVΔG-tagBFP. In contrast, no signal was observed for VSVΔG-minispike-eGFP infected cells with COVID-19 ELISA-negative human control sera (see S2 Fig for a representative serum). Similarly, RABV replicon-expressed minispike was specifically stained at the cell surface (Fig 4B). Interestingly, while the patient sera recognized the native minispike protein as expressed by VSV and RABV replicons, they did not react effectively with reduced and SDS-denatured protein in Western blots (Fig 4C). This indicated that the majority of the available human COVID-19 serum antibodies can only bind native conformational RBD epitopes.

In summary, the results showed that the transmembrane minispike protein expressed from recombinant rhabdoviruses is well recognized by conformational antibodies made in response to natural SARS-CoV-2 infection and that it largely mimics the conformational landscape of the natural SARS-CoV-2 S RBD. We reasoned that rhabdovirus replicons encoding chimeric minispike genes therefore represent promising and safe COVID-19 vaccine candidates.

### A single dose of VSVΔG-minispike-eGFP is sufficient for induction of SARS-CoV-2 neutralizing antibodies

**Fig 5:**
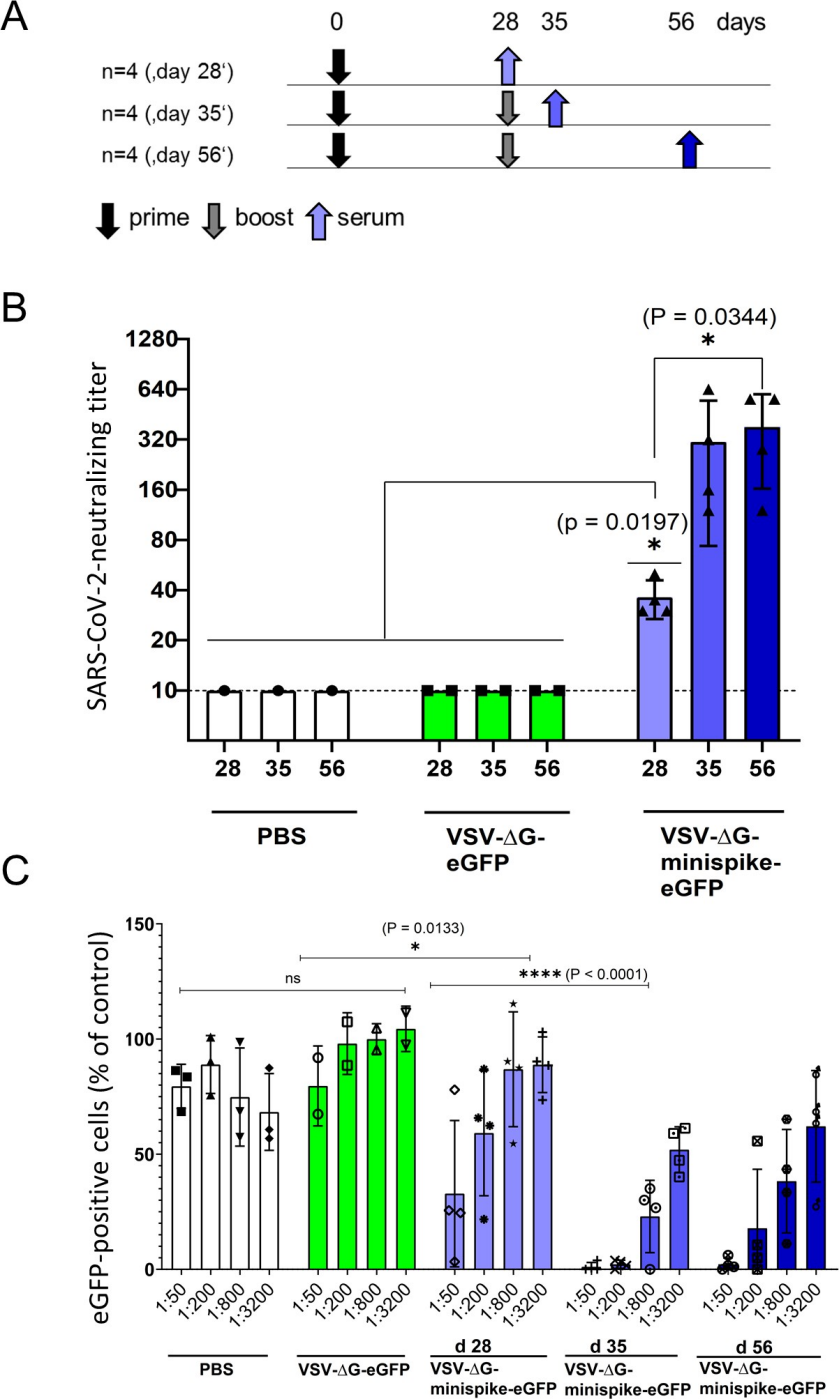
Vaccination with VSVΔG-minispike-eGFP elicits potent SARS-CoV-2 neutralizing antibodies. (A) Immunization Scheme. BALB/c mice were immunized i.m. with 1x10^6^ infectious units of VSV G-complemented VSVΔG-minispike-eGFP and controls including VSV G-complemented VSVΔG-eGFP, or PBS. Twenty-eight days after immunization serum was collected from 4 vaccinated mice, while 8 mice received an i.m. boost immunization with the same dose of virus. (B) Serum neutralization tests performed with a clinical isolate of SARS-CoV-2. The neutralizing titer of sera from vaccinated and control mice as indicated is expressed as the reciprocal of the highest dilution at which no cytopathic effect was observed. Each point represents data from one animal at the indicated time points. The bars show the mean from each group and the error bars represent standard deviations. Significant neutralizing activity was observed in mice receiving only a prime vaccination (day 28, light blue). A boost immunization further significantly enhanced neutralizing titers (days 35 and 56). (C) Neutralization of VSVΔG(S) pseudotype viruses by individual mouse sera. Mouse sera collected on day 28 (receiving prime immunization only) or at 35 and 56 days (receiving prime and boost immunization) were serially diluted as indicated and analyzed for neutralization VSV(S) pseudotype particles. GFP-encoding pseudotype virions were incubated with increasing dilutions of mouse sera or medium control before infection of VeroE6 cells. The graph shows percentage of GFP-positive cells in relation to medium controls (100%) and in dependence of dilution. Data points represent the average of three technical replicates, bars indicate standard deviation, and statistical significance was determined by one-way ANOVA.

To assess the suitability and the sufficiency of a single round VSVΔG minispike replicon to elicit an immune response, we immunized BALB/c mice with VSVΔG-minispike-eGFP (G) by intramuscular (i.m.) administration. As advised by the above results, virus stocks produced under limiting (6 hrs) VSV G complementation were used, to prevent excess formation of non-viral G vesicles. Four mice received a single dose of 1x10^6^ infectious particles, while 8 mice received an additional boost with the same virus preparation and dose 28 days following prime vaccination. As controls, mice immunized the same way with VSVΔG-eGFP (VSV G) (n = 2 for each condition) or with PBS (n = 1 for each condition) were used. The 4 mice receiving only prime vaccination were sacrificed at day 28, and 4 boosted mice each at day 35 (n = 4) and day 56 (n = 4), to collect serum (Fig 5A).

Virus neutralization assays were performed with a SARS-CoV-2 virus isolate from Wetzlar, Germany [23]. Notably, all 4 mice immunized only once developed detectable titers of SARS-CoV-2 neutralizing antibodies in the range of 1:20–1:40 dilutions (Fig 5B). Boost vaccination further increased neutralizing titers to 1:160–1:640.

For verification of the notable neutralizing titers after prime immunization in an independent assay, we also produced VSV particles pseudotyped with a functional S protein, VSV-eGFP-ΔG-GLuc (SΔC19). Neutralization assays confirmed the induction of significant levels of S-neutralizing antibodies in mice receiving a single prime vaccination and further enhancement of neutralization activity by boost immunization (Fig 5C).

**Fig 6:**
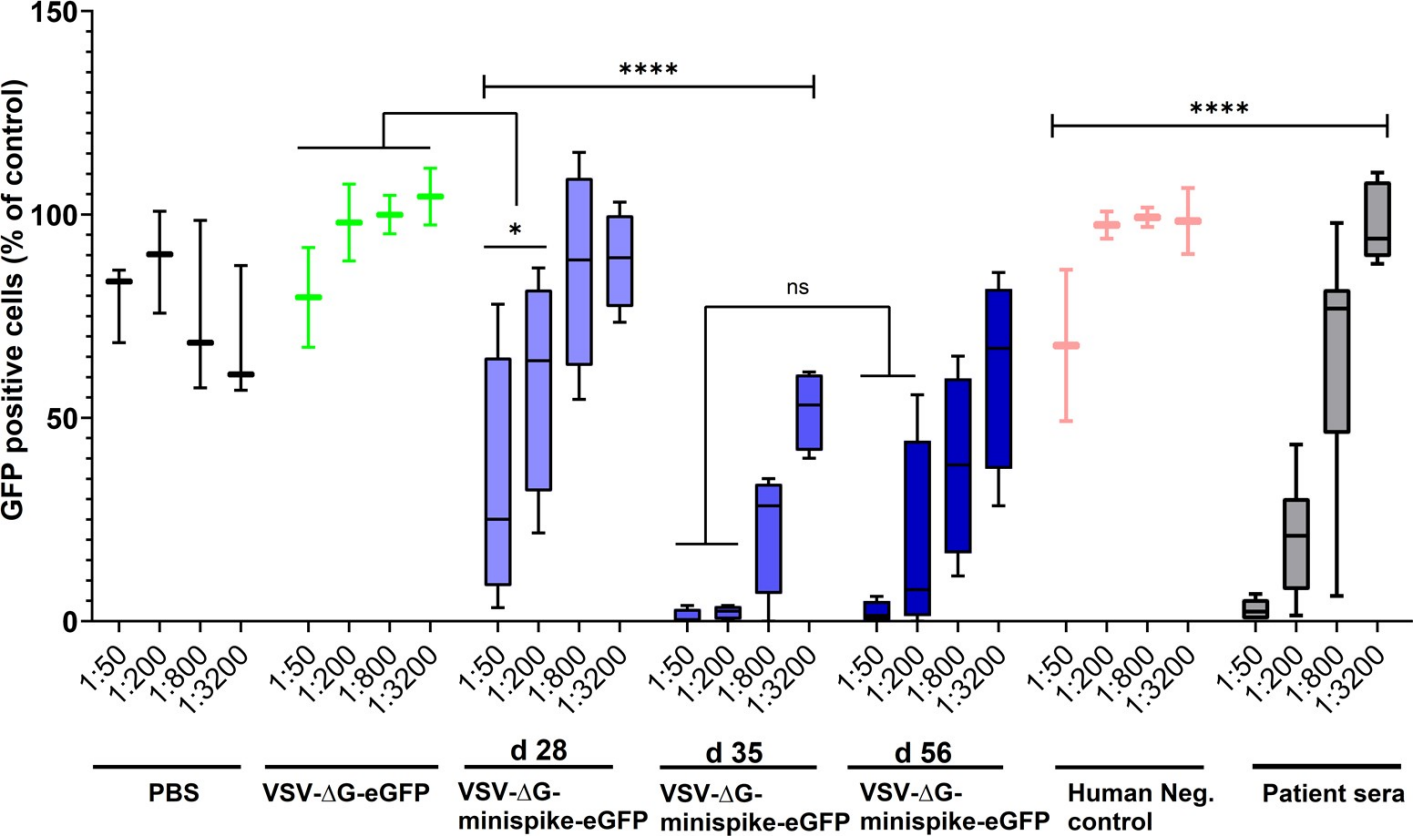
Similar virus-neutralizing titers in vaccinated mice and COVID-19 patients. VSVΔG(S) neutralization activity of sera from vaccinated mice and human immune sera tested positive for S antibodies by ELISA were compared. The graph shows percentage of GFP-positive cells in relation to medium controls and in dependence of dilution. All ELISA-positive human sera revealed VSV(S)-neutralizing activity (see S2 Fig) and are included in the grey boxes showing activity at the indicated dilutions. Primed mice (d28) exhibited neutralizing activity comparable to those of human patients, while boosted mice (d35 and d56) exhibited superior activity. Bottom and top of each box represent the first and third quartiles respectively. Whiskers represent the lowest and highest data points of the lower and upper quartile respectively. Student’s t-test and One-way ANOVA were performed to determine statistical significance.

To directly compare the neutralizing activities of sera from vaccinated mice and from COVID-19 patients, VSV-eGFP-ΔG-GLuc (SΔC19) neutralization assays were employed. Even sera with low ELISA IgG ratios revealed a marked neutralizing capacity (S3 Fig). Most intriguingly, the group of mice immunized only once (boxes labeled d 28 in Fig 6), developed neutralizing antibodies with a capacity almost equal to those of the group of COVID-19 patients (grey boxes), illustrating a powerful induction of humoral immunity by vaccination with the single round VSVΔG-minispike-eGFP replicon. Boost immunization further enhanced neutralizing titers to exceed those of patients (Fig 6).

### K18-hACE2 mice are protected from SARS-CoV-2-induced respiratory disease after a single immunization

**Fig 7:**
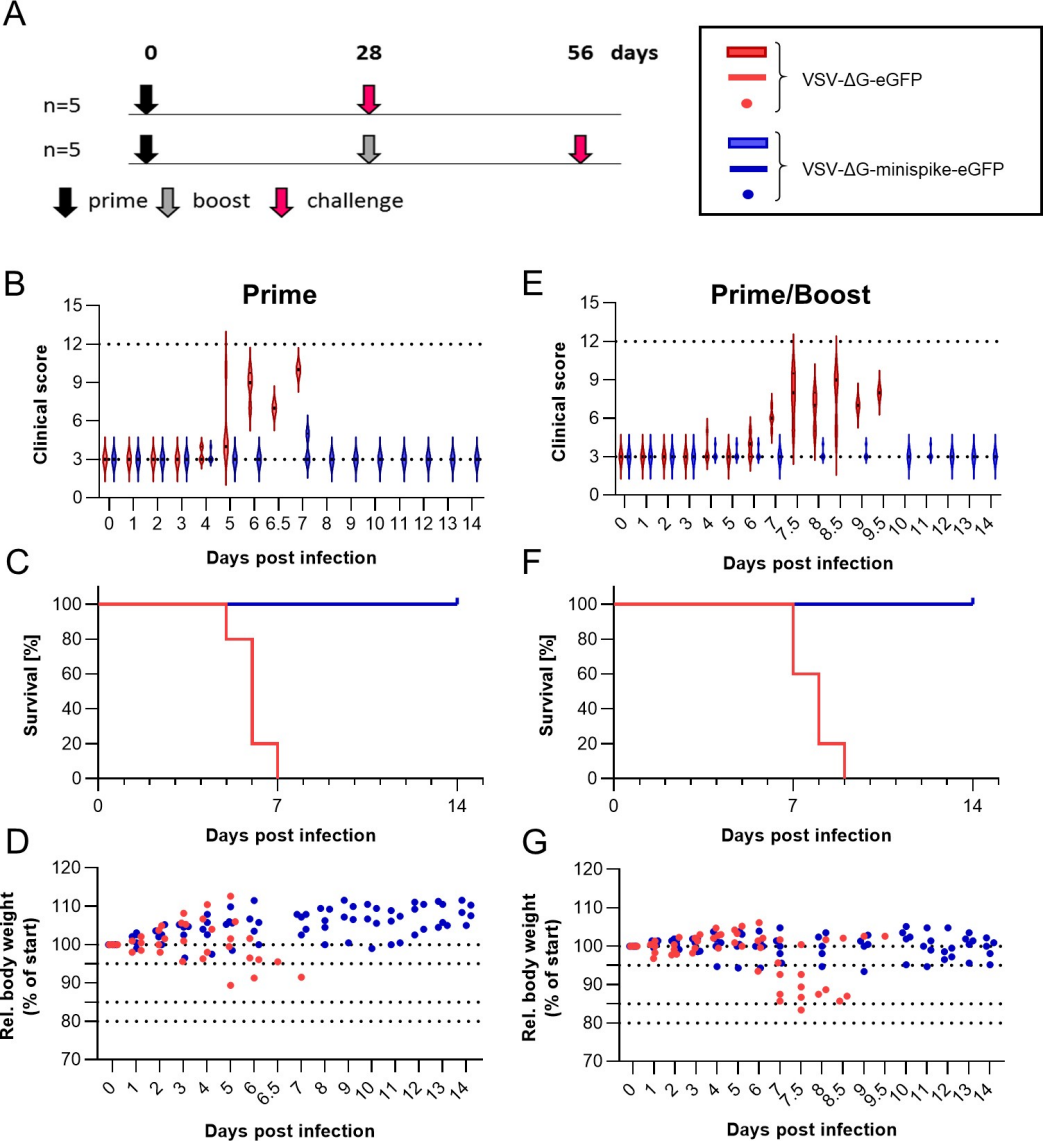
Protection from disease. (A) Immunization and challenge schematic. C57BL/6 K18-hACE2 mice (5 per group) were immunized (1x10^6^ ffu intramuscularly) once (prime, black arrow) or twice (boost, grey arrow) four weeks apart with either VSV-ΔG-minispike-eGFP (indicated in blue in panels B-G) or VSV-ΔG-eGFP (indicated in red in panels B-G) and challenged with 1x10^4^ TCID_50_ SARS-CoV-2 (Wetzlar isolate) administered intranasally four weeks after the last immunization. Mice were monitored daily for development of disease for 14 days. (B-D) Evaluation of clinical disease of challenge after prime immunization. (E-G) Evaluation of clinical disease of challenge after prime/boost immunization. (B and E) Clinical score development assessed by body weight loss, general appearance, and behavior. 3: healthy; 4–6: mild disease; 7–9: severe disease; 10–12: moribund. (C and F) Survival plots. (D and G) Body weights of individual mice relative to the weight at challenge infection. Dotted lines indicate limits of clinical scores (>95%: score = 1, 85–95%: score = 2; 80–85%: score = 3; <80%: score = 4).

To assess the protective capacity of the VSV replicon we used transgenic K18-hACE2 C57BL/6 mice, which were previously shown to develop respiratory disease resembling severe COVID-19 [76]. Five mice each were immunized as before with VSVΔG-minispike-eGFP or VSVΔG-eGFP control and challenged intranasally with 10^4^ TCID_50_ of SARS-CoV-2 Wetzlar, either following prime immunization or homologous boost immunization (Fig 7A). Mice immunized with the VSVΔG-eGFP control developed respiratory disease beginning as early as day 5 after infection (Fig 7B and 7E), which progressed over the following 3–4 days, and animals ultimately succumbed to disease 6–9 days after infection (Fig 7C and 7F). These animals lost only approximately 10–15% of their initial weight (Fig 7D and 7G), which indicates that they experienced a largely respiratory syndrome. In contrast, mice immunized with VSVΔG-minispike-eGFP experienced no clinical signs of disease (Fig 7B and 7E), and all animals survived the infection (Fig 7C and 7F) with little to no weight loss during the study (Fig 7D and 7G). This demonstrates the protective power of the VSVΔG-minispike-eGFP replicon vaccine, since a single immunization prevented the development of lethal COVID-19 respiratory disease.

## Discussion

Vaccines are used in healthy populations, therefore the highest safety standards have to be applied. Front-runner COVID-19 vaccines employ obviously innocuous mRNA delivery for expression of the prefusion-stabilized form of the S antigen [1,3] or replication incompetent adenoviruses [4]. Auspiciously, these combinations turned out to be safe, and hold great promise in containing the pandemics. Many proposed COVID-19 vaccine candidates, however, employ unmodified S protein, existing in pre- and postfusion forms and/or are based on replication competent viruses, including VSV, which is a prime vector platform for emerging diseases and cancer.

Here, we used a structure-guided approach to generate a VSV replicon vaccine meeting the requirements in terms of both virus safety and antigen harmlessness, as well as in efficacy. Our results illustrate that a small antigen, the RBD of SARS-CoV-2, if presented in the form of the present chimeric minispike protein from a safe, spreading-deficient single round biosafety level 1 rhabdovirus replicon is sufficient to elicit high levels of neutralizing antibodies. Most remarkably, a single immunization proved to be sufficient to protect SARS-CoV-2 permissive animals from lethal disease.

Assessment of antibody responses to different betacoronaviruses has recently underlined that the SARS-CoV-2 RBD is the prime target for COVID-19 vaccination. While SARS-CoV-1 and MERS-CoV S proteins encode a number of VNA epitopes located outside of the RBD, the SARS-CoV-2 RBD seems to account for almost all human antibodies with potent neutralization capacity [22,32–34,77], with rare exceptions [78,79]. Furthermore, presentation of the antigen is key for the success of immunization. While this work was in progress, data on various S protein constructs became available. While soluble monomeric RBD protein suffered from limited immunogenicity, a tandem repeat single chain construct enhanced immunogenicity [80]. A soluble trimeric RBD, as applied in BNT162b1 mRNA clinical trials, showed very promising immunogenicity including stimulation of antibodies and T cell responses [81,82]. In addition to trimerization, membrane anchoring seems to further improve immunogenicity, as transmembrane anchored prefusion-stabilized full-length S protein was reported to elicit higher VNA levels than corresponding secreted constructs [1,83]. Both in terms of immunogenicity and potential association of circulating SARS-CoV-2 S1 subunit with enhanced blood clotting [84], use of a small membrane-anchored antigen is rational.

Here we used a previously applied strategy to present a trimeric transmembrane RBD in the form of a chimeric rabies virus/SARS-CoV-2 minispike. The discrete folding into a globular structure of the RBD [27,28,57] called for its combination with a rhabdovirus stem-anchor construct we have previously identified as suitable for presentation of a structurally intact protein domain (dsRED) on the surface of cells and on RABV particles [59]. The antigenic properties of the RBD in the context of the minispike remains similar to those in natural S protein, as initially indicated by binding of COVID-19 patients’ IgG and Mab CR3022 to cells expressing the minispike construct (Figs 1 and 4). Actually, we were initially astonished by the strong immune fluorescence signals, but in the meantime extensive characterization of natural human and animal monoclonal antibodies has revealed multiple, independent conformational epitopes in the RBD [22,32–34,62,85,86]. The simultaneous targeting of distinct RBD antigenic sites is of relevance not only for the efficiency of a vaccine but also in the light of emergence and spread of SARS-CoV-2 variants resistant against individual antibodies [54,86]. Ongoing screening of rat monoclonal antibodies generated in response to VSVΔG-minispike-eGFP will reveal whether the chimeric minispike presents a full complement of natural RBD epitopes.

The minispike is presented copiously at the cell surface membrane, and in addition is incorporated into rhabdovirus VLPs, or mosaic viruses in the presence of G, as confirmed by immune fluorescence of cells, immune blot, and cryo-EM of virions. Reflecting the previous observations, that RABV and VSV G protein trimers are rather instable [71,87,88], we could not immediately demonstrate a trimeric form of the minispike on the cell surface. In the context of viral envelopes, however, in which the internal RNP and matrix protein layers determine organization [70,72,88,89], trimeric G spikes form highly ordered paracrystalline arrays. It was suggested that the repetitive arrangement of G epitopes as observed in VSV is responsible for stimulating a very strong antibody response, by crosslinking of B cells via receptors, and possibly by contribution of T cell-independent mechanisms [90,91]. VLPs in general are potent immunogens, and some VLPs may be transported to local lymph nodes to promote immune responses [92]. We assume that the non-infectious minispike VLPs as generated here are synergizing with cell membrane expressed antigen, although quantification of their exact contribution to the overall immune response will require further experimentation with purified VLPs.

The excellent immunogenicity of the minispike in the context of a single-round, G-deficient VSV vaccine was illustrated by induction of high levels of SARS-CoV-2 neutralizing antibodies in mice. VNA activities equaling those of COVID-19 patients were detectable in animals receiving only a single i.m. dose of vaccine, and boost vaccination with the identical virus in the same hind leg further boosted VNA activity to levels superior to those of COVID-19 patients. VSV and RABV infections are known to induce a strong Th1 biased antiviral and anticancer immune response [93,94]. This holds also true for VSVΔG-minispike-eGFP vaccination, as indicated by preliminary results from rats. More than 95% of S positive IgG hybridomas produced immune globulins of the IgG2 subclass, while IgG1 was only sporadically observed, thus reflecting strong Th1 immune response. Finally, complete protection of K18-hACE2 mice from SARS-CoV-2 disease after a single immunization confirmed both safety and efficacy. This qualifies VSVΔG-minispike constructs as promising vaccine candidates meriting further investigation. In particular, it will be interesting to reveal whether vaccination will be sufficient to prevent transmission of the virus in suitable animal models.

While the chimeric minispike construct as described here appears to be immediately suitable in any genetic vaccine approach, including the auspicious mRNA platforms [81], its full potential is accomplished in the context of the highly flexible rhabdovirus vector system, which integrates antiviral innate and adaptive immune responses. As shown here, the VSVΔG replicon complemented with little VSV G protein to mediate infection of muscle cells is highly effective in SARS-CoV-2 S RBD antigen expression after i.m. application, and intraperitoneal (i.p.) administration is supposed to be similarly effective [43]. Boost immunizations with the same virus led to strong increase in VNA titers, allowing both homologous and heterologous boost strategies. While results for RABVΔG-based minispike vaccines are not yet available, both VSV and RABV are amenable to envelope switching, such that pseudotyping of rhabdovirus minispike replicons with a variety of heterologous G proteins is feasible. While generation of VSV replicons expressing multiple and variant RBDs is practicable (Fig 2), the option of envelope switching may be valuable for performing boost immunizations against emerging SARS-CoV-2 variants, or to achieve appropriate immune responses in elderly or immunocompromised individuals. Moreover, RABVΔG or VSVΔG minispike vectors complemented with the G protein of widely used RABV strains like SAD offer the intriguing possibility of oral immunization, in the context of prime or boost regiments.

## Methods

### Ethics statement

Mouse immunization studies were carried out in the animal housing facility of the Paul-Ehrlich-Institute, in compliance with the regulations of German animal protection laws and authorized by the responsible state authority (V54-19c20/15-F107/1058) and V54-19c18-F107/2006). Diagnostic use of anonymous patient sera was approved by the Ethics Committee of the Medical Faculty of the LMU.

### Cells

HEK293T and VeroE6 (ATCC) were maintained in DMEM Medium (GIBCO) containing 10% fetal bovine serum, 1% L-Glutamine (GIBCO) and 0,5% Pen. Strep (GIBCO). BHK-G43 cells [65], kindly provided by Georg Herrler, and BSR-MG-on cells [95] were maintained in GMEM media containing 10% fetal bovine serum, 0,5% Pen/Strep, 1% MEMs/NEAAs and 19,5mL tryptose phosphate broth (Thermo Fisher). VSV G expression in BHK-G43 cells was induced by adding 10^−9^ molar mifepristone 3 hours prior to infection, and RABV G in MG-on cells by adding 1 μM doxycycline. All cells were maintained at 37°C under 5% CO_2_.

### Construction of cDNAs

NCBI Reference Sequence NC_045512.2 of nCoV, Wuhan isolate 1, was used to synthesize human codon optimized cDNAs encoding full length HA-tagged spike (S-HA), and minispike (Thermo Fisher GeneArt). The minispike construct comprised S residues 314–541, QTSN…KCVNF fused via a GSG linker to the stem-anchor construct of SAD G described in [59]. Constructs were inserted into pCR3 for analysis of protein expression in transfected HEK293T cells and for further subcloning in RABV and VSV replicon cDNA. For production of VSVΔG(S) pseudotype viruses, we used an S-HA derived construct, pCG-S-ΔC19, kindly provided by Christian Buchholz, PEI. Plasmids for expression of wt S Protein and S variants included pCG-nCoV-S, pCG-nCoV-SΔC, and pCG-nCoV-S-V5, kindly provided by Konstantin Sparrer and Caterina Prelli Bozzo.

### Construction and rescue of recombinant rhabdoviruses

To obtain recombinant replication-competent VSVs, an infectious plasmid clone of VSIV, pVSV-eGFP [39] (kindly provided by Jack Rose) was used to insert minispike cistrons or exchange the eGFP cassette with single or multiple copies of the minispike cistron. To yield G gene-deleted VSV replicons encoding RBD minispike the VSV G gene was replaced with minispike cassettes. To generate VSV-eGFP-ΔG-GLuc, VSVeGFPΔG (addgene #31842, kindly provided by C. Cepko) was used to insert a cistron encoding Gaussia Luciferase (GLuc) between G and L genes. pVSVΔG-4BFP2 was obtained from I. Wickersham via addgene, (#64101). Virus rescue was performed in HEK293T cells transfected with the viral cDNA plasmids directing T7 RNA polymerase-driven transcription of viral antigenome (+) RNAs from a T7 promoter along with expression plasmids encoding T7 RNA polymerase and VSV helper proteins N, P, and L (pCAG-T7, -N, -P, -L; addgene #59926, #64087, #64088, #64085, respectively, all provided by I. Wickersham). Virus stocks of VSVΔG constructs were produced in HEK293T cells transfected with pCAGGs-VSV G, or BHK-G43 and concentrated by ultracentrifugation over a 20% sucrose cushion in a SW32 rotor at 24,000 rpm and 4°C for 2h.

Recombinant cDNAs of RABVΔG expressing minispike and mNeonGreen were generated by replacement of the eGFP cassette of pHH_SADΔG-eGFP_SC with two transcription units and rescued into virus in cells providing RABV N, P, L, and T7RNA polymerase as described before [66–68]. RABVΔG replicons were propagated in MG-on cells providing SAD G [95].

### Western blots

Laemmli SDS-PAGE in 6% stacking and 10% separating Bis-Tris gels and Western blot analysis on semi-dry-blotted PVDF membranes was done as previously described [96]. Briefly, membranes were incubated with primary antibodies at 4°C overnight, and after three times washing with TBS-T incubated for 2 hrs with horseradish peroxidase-conjugated secondary antibodies at room temperature. Bio-RAD Clarity Western Enhanced Chemiluminescence (ECL) Substrate kit was used for detection in a Fusion Fx7 imaging system.

### Microscopy

For live cell imaging, VeroE6 or HEK293T cells were seeded into poly-D-lysine (Millipore-Sigma)-coated multiwell plates one day prior to infection with VSV replicons at the indicated MOIs or plasmid transfection by lipofection, respectively. Infected cells were incubated overnight at 32°C. Minispike was detected by incubation with human COVID-19 patient sera or the serum of a healthy donor diluted 1:300 in DMEM fluorobrite for one hour at 37°C and subsequent staining with anti-Human IgG (H+L) AlexaFluor555 (1:2,000 in DMEM Fluorobrite, 1h, 37°C) and imaged after washing with DMEM fluorobrite. For fixation and permeabilization, infected or transfected cells were washed once with PBS, treated with 4% PFA for 20 min at room temperature, and permeabilzed by 0.05% Saponine. After blocking with 5% bovine serum albumin (BSA) in PBS for 1h at room temperature, and three times washing, cells were incubated with COVID-19 patient sera and HCA-5 rabbit peptide serum in PBS with 1% BSA over night at 4°C. The cells were then washed three times with PBS and incubated with anti-Human IgG (H+L) AlexaFluor488 and anti-Rabbit IgG (H+L) AlexaFluor555 for one hour at room temperature. After 3 washing steps, cells were imaged on a Leica DMi8 microscope equipped with LED405 (blue), GFP (green), TXR (red) and Cy5 (far red) filter cubes.

### Cryo-electron microscopy

Concentrated preparations of rhabdovirus pseudotype particles were added to glow discharged Quantifoil 200 mesh 2/1 holy carbon copper grids in the presence of Aurion protein A 10nm gold beads. Vitrification was performed either with a manual plunging unit or a FEI Vitrobot. Grids were analyzed in a FEI Glacios or a FEI Talos Arctica operated at 200kV and bidirectional or dose symmetric tilt series were acquired with a FEI Falcon 2 direct electron detector. Tomograms were subsequently reconstructed with etomo and visualizsed with 3dmod [97]. Image segmentation was conducted in Amira (ThermoFisher). Surface representations and molecular graphics images were produced using the UCSF Chimera package [98].

### Animal experiments

Immunogenicity of our vectors was initially evaluated in 11–19 weeks old female BALB/c mice. Challenge studies after immunization were carried out in 6–20 weeks old female, transgenic C57BL/6 mice expressing human ACE2 under control of the keratin-18 promoter [99] (K18-hACE2, Jackson strain no. 034860). Mice were purchased from Charles River.

BALB/c mice received one or two intramuscular injections of 1x10^6^ ffu of either VSVΔG-minispike-eGFP (VSV G), or VSVΔG-eGFP (VSV G) dissolved in 30 μl PBS, or an equal volume of PBS alone, four weeks apart (prime or prime/boost). For evaluation of immunogenicity, mice receiving only a single dose of vaccine were sacrificed on day 28, mice from the prime/boost group were sacrificed on days 35 or 56. The mice were anesthetized by intraperitoneal injection of 100 mg/kg body weight ketamine and 10 mg/kg body weight xylazine and exsanguinated retroorbitally or by cardiac puncture. Whole blood was collected in Z-gel containing tubes (Sarstedt). Serum was separated by centrifugation at 14,000 g for 10 min at 4°C and stored at -20°C.

For the challenge studies, immunized K18-hACE2 mice were immunized as before and transferred to a BSL3 facility on day 28 post immunization (prime group) or day 56 post immunization (prime/boost group). Animals were anesthetized by intraperitoneal injection of 100 mg/kg body weight ketamine and 4 mg/kg body weight xylazine and intranasally infected with 10^4^ TCID_50_ of SARS-CoV-2 (Wetzlar isolate), kindly provided by Eva Friebertshäuser, in a total volume of 10 μl. Animals were evaluated daily for weight loss, behavior, and appearance, and received a score between 1 and 4 in each category. A clinical score was calculated as the sum of each individual value. Mice were euthanized when they reached a score of 4 in at least one of the three categories.

### Virus neutralization assays

SARS-CoV-2 neutralizing antibody titers were determined by mixing serial dilutions of serum collected from mice at the indicated time points with 10^2^ TCID_50_ of SARS-CoV-2 (Wetzlar isolate). Virus and serum dilutions were incubated at 37°C for 20 min before 50 μl of VeroE6 cells were added to each well. After incubation for 3 days at 37°C, cell monolayers were stained with PBS containing 4% paraformaldehyde (PFA) and 1% crystal violet. The neutralizing titer is expressed as the reciprocal of the highest dilution at which no cytopathic effect (CPE) was observed.

Neutralization of VSV (S) pseudotyped viruses were performed as follows: HEK293T cells transfected with pGC-SΔC19 (obtained from Christian Buchholz) for one day were infected with VSV G-complemented VSV-eGFP-ΔG-GLuc at a MOI of 1. After 3 hrs incubation, excess input virus was removed by thorough washing. After incubation for 24h, supernatant was collected, and S pseudotype virions concentrated by ultracentrifugation through a sucrose cushion, resuspended in PBS and titrated on VeroE6 cells in the presence of VSV-neutralizing hybridoma supernatant (I1-Hybridoma, ATCC CRL-2700) to block residual input G-containing virus. Briefly, VeroE6 cells were seeded at a density of 1x10^4^ cells per well in 96 well-plates, and incubated with 10^2^ ffu of S pseudotype viruses in the presence of I1 supernatant, and with cell culture medium as a control, or increasing dilutions of mouse or human sera, as indicated, in a total volume of 100 μL. Infectious units were determined after one-day incubation by manual counting of fluorescent cells in triplicate experiments.

### Data representation and statistical analysis

Statistical analyses were performed using GraphPad Prism version 8.4.3. Unless otherwise stated, data are from at least three technical replicates. Statistical significance was calculated using 2-tailed Student t-test or one-way ANOVA; results are indicated in figures (* p = 0.05; ** p<0.01; *** p<0.001; **** p<0.0001; ^ns^ not significant).

## Supporting information

S1 Fig (related to Fig 3). (A) Cryo-electron tomograms (i) of RABV SAD (left panels; *G only*) and of minispike-encoding RABV replicons generated in the presence of the autologous RABV G protein (middle panels, *G + minispike*) or in the absence (*minispike*; right panels) are shown. Magnification of the indicated areas is shown in (ii). (iii) Representation of densities surrounded by the viral envelope (blue) and the glycoprotein layer in purple. (B) Length distribution of surface proteins on RABV particles.
(TIF)

S2 Fig (related to Fig 4). **Recognition of VSV-expressed minispike by patient sera.** VeroE6 cells were infected with VSV-ΔG-bimini overnight at 32°C, fixed in 4% PFA and permeabilized with 0.1% Saponine followed by incubation with different S ELISA-positive sera of COVID-19 patients, and HCA-5 as a control for minispike expression. In contrast to a human negative control serum, all patient sera stained cells expressing minispike with anti-human IgG (H+L) Alexa 488 (green). ToPro3 (magenta) was used to counterstain nuclei.
(TIF)

S3 Fig (related to Fig 6). **Characterization of COVID-19 patient sera.** (A) ELISA IgG ratio of sera (B) VSV(S) neutralizing activity of human sera. Graph shows reduction of ffu of VSVeGFP-ΔG-GLuc S pseudotype viruses after incubation with sera at the indicated dilutions. (C) Comparison of ELISA titers and neutralizing activity at 1:200 dilution.
(TIF)
