# Supplementary figures and images for: Safe and effective two-in-one replicon-and-VLP minispike vaccine for COVID-19: Protection of mice after a single immunization
Source: PLoS Pathog. 2021 Apr 21;17(4):e1009064. doi: 10.1371/journal.ppat.1009064 (PMC8092985; doi:10.1371/journal.ppat.1009064)

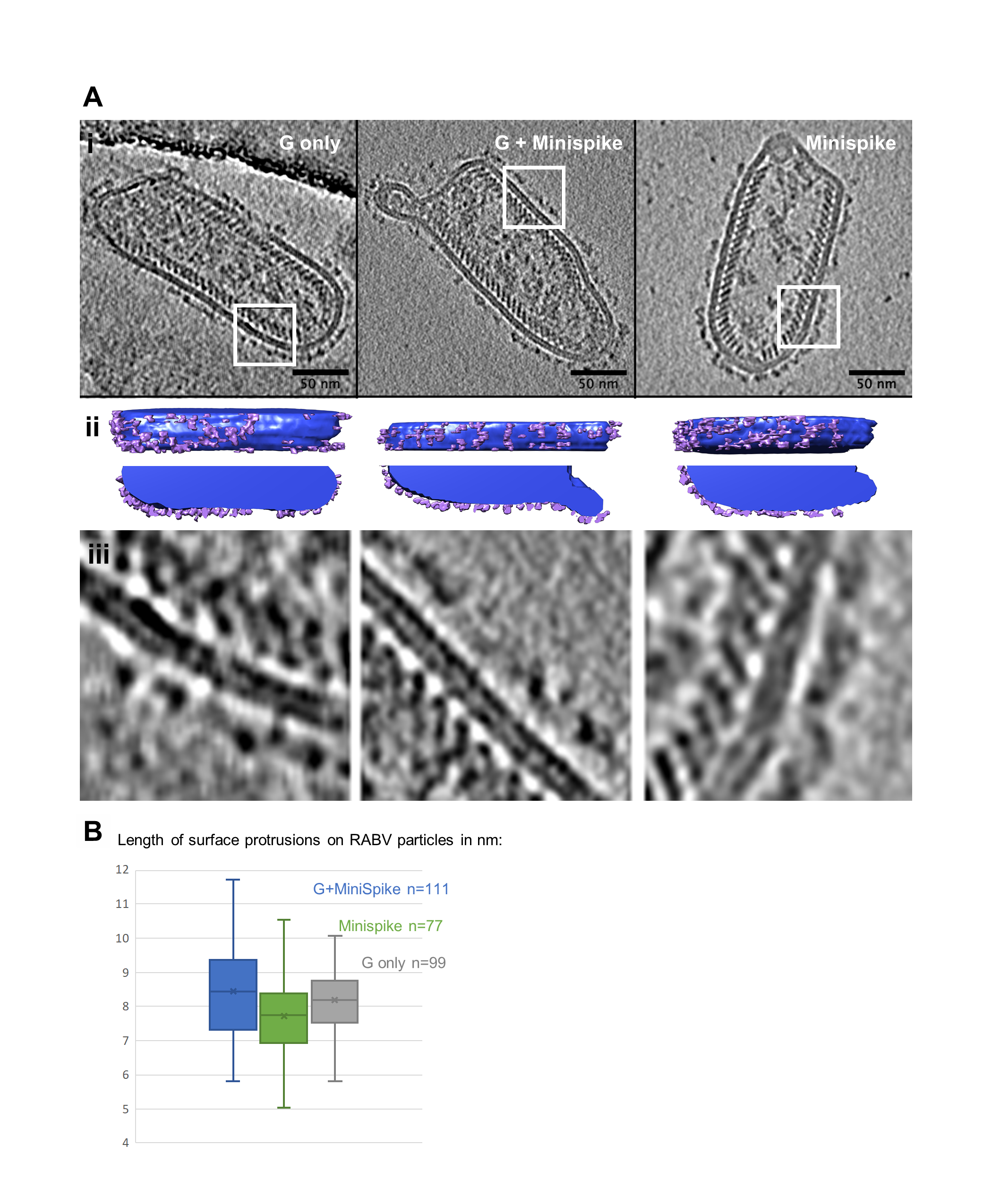

Supplement: S1 Fig — (A) Cryo-electron tomograms (i) of RABV SAD (left panels; G only) and of minispike-encoding RABV replicons generated in the presence of the autologous RABV G protein (middle panels, G + minispike) or in the absence (minispike; right panels) are shown. Magnification of the indicated areas is shown in (ii). (iii) Representation of densities surrounded by the viral envelope (blue) and the glycoprotein layer in purple. (B) Length distribution of surface proteins on RABV particles. (TIF) [file ppat.1009064.s001.TIF]

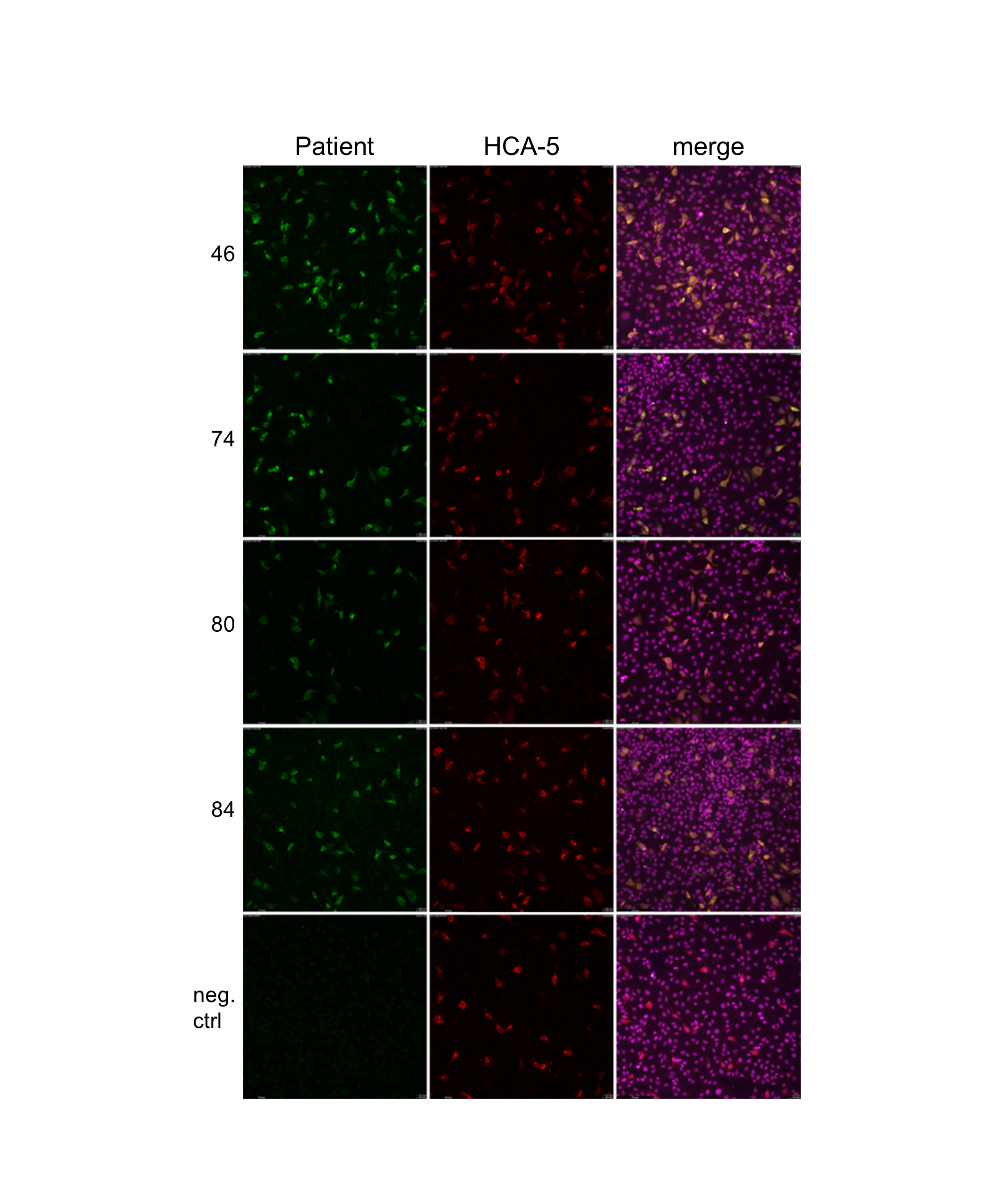

Supplement: S2 Fig — Recognition of VSV-expressed minispike by patient sera. VeroE6 cells were infected with VSV-ΔG-bimini overnight at 32°C, fixed in 4% PFA and permeabilized with 0.1% Saponine followed by incubation with different S ELISA-positive sera of COVID-19 patients, and HCA-5 as a control for minispike expression. In contrast to a human negative control serum, all patient sera stained cells expressing minispike with anti-human IgG (H+L) Alexa 488 (green). ToPro3 (magenta) was used to counterstain nuclei. (TIF) [file ppat.1009064.s002.TIF]

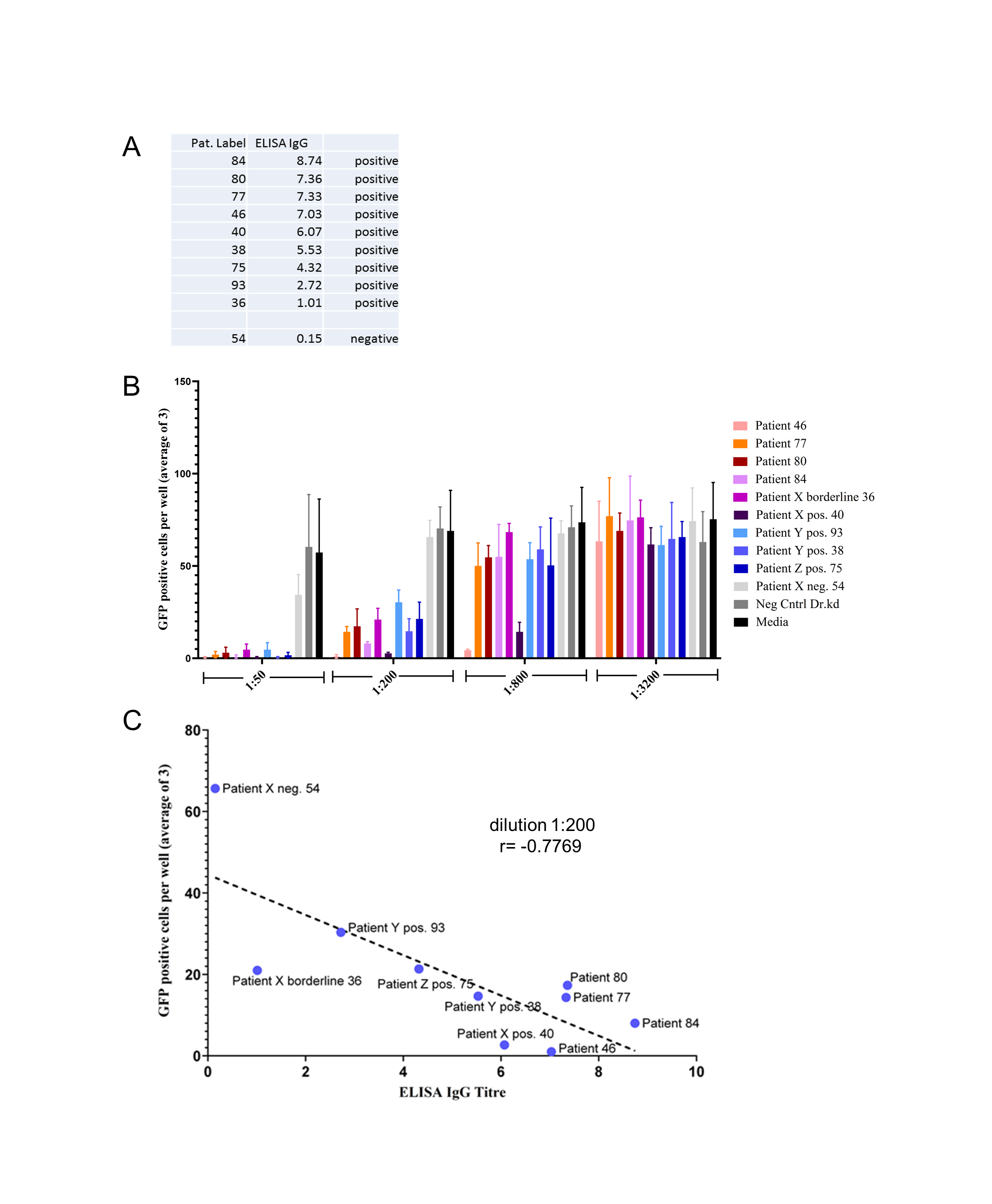

Supplement: S3 Fig — Characterization of COVID-19 patient sera. (A) ELISA IgG ratio of sera (B) VSV(S) neutralizing activity of human sera. Graph shows reduction of ffu of VSVeGFP-ΔG-GLuc S pseudotype viruses after incubation with sera at the indicated dilutions. (C) Comparison of ELISA titers and neutralizing activity at 1:200 dilution. (TIF) [file ppat.1009064.s003.TIF]
